# The effects of metabolism on the immune microenvironment in colorectal cancer

**DOI:** 10.1038/s41420-024-01865-z

**Published:** 2024-03-07

**Authors:** Xingzhao Chen, Zhiyuan Ma, Zhiqiang Yi, Enqin Wu, Zhengye Shang, Biguang Tuo, Taolang Li, Xuemei Liu

**Affiliations:** 1 Department of Gastroenterology, Digestive Disease Hospital, Affiliated Hospital of Zunyi Medical University, Zunyi, Guizhou Province China; 2 Department of General Surgery, Affiliated Hospital of Zunyi Medical University, Dalian Road 149, Zunyi, 563000 China

**Keywords:** Colorectal cancer, Immune evasion, Cancer metabolism, Cancer microenvironment

## Abstract

Colorectal cancer (CRC) is a malignancy that is widely prevalent worldwide. Due to its unsatisfactory treatment outcome and extremely poor prognosis, many studies on the molecular mechanisms and pathological mechanisms of CRC have been published in recent years. The tumor microenvironment (TME) is an extremely important feature of tumorigenesis and one of the hallmarks of tumor development. Metabolic reprogramming is currently a hot topic in tumor research, and studies on this topic have provided important insights into CRC development. In particular, metabolic reprogramming in cancer causes changes in the composition of energy and nutrients in the TME. Furthermore, it can alter the complex crosstalk between immune cells and associated immune factors, such as associated macrophages and T cells, which play important immune roles in the TME, in turn affecting the immune escape of tumors by altering immune surveillance. In this review, we summarize several metabolism-related processes affecting the immune microenvironment of CRC tumors. Our results showed that the immune microenvironment is regulated by metabolic reprogramming and influences the development of CRC.

## Facts

- The altered TME, influenced by metabolic reprogramming, affects the complex crosstalk between immune cells and immune factors, such as macrophages and T cells, leading to immune escape and tumor development.
- Metabolic reprogramming in colorectal cancer (CRC) is driven by alterations in several key signaling pathways, such as the PI3K-Akt-mTOR pathway.
- Metabolic alterations in CRC, including increased aerobic glycolysis (Warburg effect) and altered lipid metabolism, contribute to tumor growth, invasion, and metastasis.
- Targeting metabolic vulnerabilities in CRC, such as inhibiting specific metabolic enzymes or transporters, holds promise as a therapeutic strategy to enhance treatment efficacy.

## Open questions

- How does metabolic reprogramming in CRC specifically affect the recruitment and polarization of immune cells within the tumor microenvironment?
- Can modulation of the gut microbiota through dietary interventions or the use of probiotics alter the metabolic landscape in CRC and improve treatment outcomes?
- Are there metabolic biomarkers that can predict the response to specific therapies in CRC patients, and can they be used for personalized treatment selection?
- How do metabolic alterations in CRC influence the efficacy and toxicity of chemotherapy and immunotherapy, and can combination therapies targeting both metabolism and immune checkpoints improve patient outcomes?

## Introduction

**Fig. 1:**
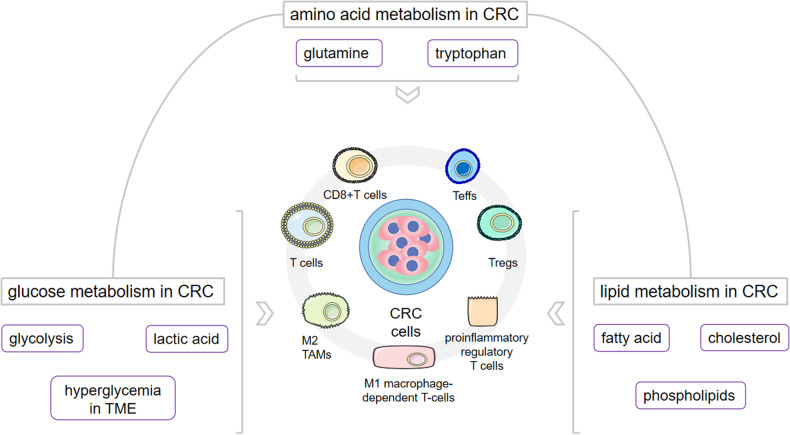
The three metabolisms on the TME of CRC. The three major metabolisms of CRC, their specific metabolic pathways and downstream products have effects on the various immune components of TME and consequently influence the progression of CRC. TME tumor microenvironment, CRC colorectal cancer.

CRC is currently the third most common cancer and the fourth leading cause of cancer death worldwide, [1] and a wide range of complex genetic and environmental risk factors are associated with poor treatment options and poor prognosis. [2] Metabolic reprogramming is an active process controlled by oncogenes and tumor suppressors. In CRC, most of the core metabolic pathways, including the three classical metabolic pathways of glucose, amino acids and lipids, are used by cancer cells to maintain their high rate of cell division and to promote rapid growth. Currently, analysis of the TME provides important insight into the process of tumor development, [3] which refers to the cellular environment in which tumors or cancer stem cells exist. The tumor microenvironment also includes surrounding immune cells, blood vessels, the extracellular matrix (ECM), fibroblasts, lymphocytes, bone marrow-derived inflammatory cells and signaling molecules, which influence tumor progression and the response to immunotherapy. In CRC, immune cells in the TME can secrete relevant immune factors to influence cancer development; these cells in turn secrete cytokine mediators to increase the output of immune cells, creating an interesting closed loop. Recent studies have shown that alterations in various metabolic pathways and corresponding metabolites after CRC metabolic reprogramming can increase or decrease the levels of immune cells and immune factors in the TME and thus play a role in the prognosis of CRC patients. This article summarizes the changes in the TME following reprogramming of the three major metabolic pathways and metabolites involved in amino acid metabolism, glucose metabolism and fatty acid metabolism in CRC. Overall, the combined effect of reprogramming these three major metabolic pathways can facilitate CRC cell immune escape, provide a supply of energy and create an ideal environment for rapid CRC development, thus promoting CRC progression **(**Fig. 1**)**.

### Impact of amino acid metabolism on the immune microenvironment of CRC

As fundamental components of life, amino acids support cellular and organismal functions by synthesizing proteins, producing ATP, synthesizing nucleotides and participating in redox homeostasis. [4] An abundant supply of amino acids is important for maintaining cancer cell proliferation. [5] Dysregulated metabolism/catabolism of glutamine, serine and glycine, and tryptophan has been identified as a metabolic regulator that supports cancer cell growth, [6–8] acting as a substrate for various types of immune cells and exerting distinctive immune effects. In CRC, the two main components of amino acid metabolism, glutamine and tryptophan, are involved in the alteration of the TME.

#### Glutamine upregulates and participates in immune regulation and promotes CRC progression

Glutamine is the most abundant and versatile amino acid in the body, and its physiological role is to serve as a key substrate for energy production, protein synthesis, and immune function and is essential for the proliferation, activation and efficacy of various immune cells. [9, 10] Furthermore, glutamine enhances cancer cell survival and proliferation through metabolic reprogramming in the TME, [11] thereby promoting cancer progression. In CRC, the synthesis and catabolism of glutamine are reprogrammed simultaneously: 1) For the synthesis of glutamine, alanine-serine-cysteine transporter 2 (ASCT2) plays an important role. In CRC, the expression of ASCT2 is increased by the regulation of several specific highly expressed genes, such as c-Myc, which in turn leads to an increase in glutamine synthesis. [12, 13] 2) For the catabolism of glutamine, one major pathway is the conversion of glutamine to glutamate by the enzyme glutaminase. Glutamate can then be further metabolized to provide energy or serve as a precursor for other biosynthetic pathways. [14, 15] Given the high glutamine concentration due to ASCT2 upregulation, there is an immune effect on the TME. [16] By blocking glutamine in mice injected with a CRC cell line, CD8 + T cells increase the effect of using acetate as a carbon source for the tricarboxylic acid (TCA) cycle, a process supported by acyl-coenzyme A (CoA) synthase, ultimately leading to enhanced levels of CD8+ tumor-infiltrating lymphocyte proliferation, activation and lifespan and an inhibitory effect on CRC. [17, 18] Interestingly, such a phenomenon also occurs in the absence of tumors. [19] In addition, reduced levels of glutamine induce the mRNA expression of EMT transcription factors (including zeb1, zeb2 and snail2). [20] When these factors are elevated, cancer-associated fibroblasts (CAFs) sense glutamine in the environment and actively shift to regions that express high amounts of glutamine, thereby promoting ECM deposition and remodeling; extensive crosstalk with cancer cells; and EMT, invasion, metastasis and treatment resistance, which play key roles in cancer progression. [21] Interestingly and paradoxically, glutamate dehydrogenase (GDH), a key enzyme that converts glutamate to a-ketoglutarate and subsequently enters the TCA cycle, has been reported to be upregulated in CRC and strongly associated with the metastasis and invasion of colorectal cancer; for example, both glutamine and its major metabolite, glutamate, have a profound impact on the poor prognosis of CRC patients. However, to date, there are many reports on the roles of glutamine and fewer on the effects of glutamate in CRC; follow-up studies are needed to confirm the metabolic reprogramming of CRC as well as its consequences for cancer progression.

#### Two metabolic routes and immunological effects of tryptophan in CRC

**Fig. 2:**
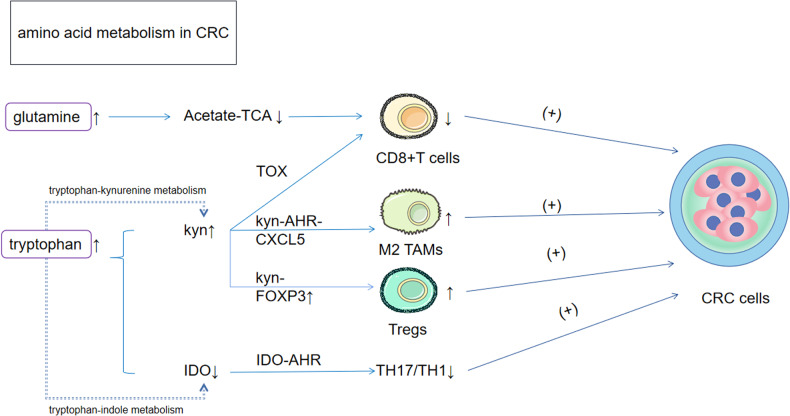
Effects of amino acid metabolic reprogramming on CRC. 1) Glutamine was upregulated in CRC by reducing the effect of acetate as a carbon source for the TCA cycle and ultimately downregulating CD8 + T cells. 2) Tryptophan is metabolized in two ways to Kyn and IDO. On one hand, Kyn affects CD8 + T cells, M2 TAMs, Tregs and ultimately promotes CRC progression via TOX, AHR and FOXP3 respectively. Reduction of IDO is also able to reduce the TH17/TH1 response through AHR, which in turn promotes the development of CRC. Kyn kynurenine; IDO indoleamine 2,3-dioxygenase 1; TAMs tumour-associated macrophages; Tregs T regulatory cells; TOX thymocyte selection-associated HMG box protein; AHR aryl hydrocarbon receptor; FOXP3 forkhead box protein.

As nutritionally essential amino acids, tryptophan and its metabolites play important roles in protein synthesis, serotonin secretion, and the regulation of mood, sleep, and immune function. [22, 23] In CRC, two important metabolic pathways involving tryptophan are reprogrammed: 1) Trp-kynurenine metabolism, which is mediated mainly by indoleamine 2,3-dioxygenase 1 (IDO1); and 2) Trp-indole metabolism, which is mediated by intestinal microbes, with the former being enhanced and the latter being attenuated. [24] In CRC, increased tryptophan (Trp) uptake mediated by Trp transporter protein upregulation leads to kynurenine (Kyn) upregulation, [25] resulting in the following immune effects: 1) Kyn interacts with the aryl hydrocarbon receptor (AHR), [26] promotes C-X-C motif chemokine ligand 5 (CXCL5) secretion and recruits M2 macrophages to enhance tumor invasiveness via EMT [27, 28]; 2) Kyn positively correlates with Forkhead box P3 (FOXP3) expression [29] and enhances tumor invasiveness via T regulatory cell (Treg) upregulation of programmed death-ligand 1 (PD-L1) expression, which enhances immune tolerance in the TME, helping CRC with immune evasion [30]; and 3) induction of CD8 + T-cell depletion through upregulation of the expression of the transcription factor Thymocyte selection-associated high mobility group box protein (TOX), [30, 31] which reduces immune efficacy against CRC. In conclusion, Kyn upregulation plays a very important role in the development of CRC and poor prognosis and has led to extensive research by investigators investigating IDO, the metabolic enzyme most relevant for the conversion of tryptophan to Kyn. [26, 32] Like Kyn and AHR, indole, which are involved in other metabolic routes of tryptophan, activates AHR but induces Treg differentiation, limits T-helper 17 (Th17) and T-helper 1 (Th1) cell responses and produces anti-inflammatory mediators, [33] which have inhibitory effects on CRC tumorigenesis. In addition, the tryptophan-derived oxazoloisoindolinone stapled peptide derived from p53-1 (SLMP53-1) can upregulate p53, [34, 35] which in turn exerts a powerful regulatory force on the TME to counteract tumor development. [36] To summarize the immune efficacy of tryptophan, the use of IDO1 as a therapeutic target could be the next key node in the fight against CRC **(**Fig. 2**)**.

### Effect of glucose metabolism on the immune microenvironment of CRC

Under aerobic conditions, normal cells use oxidative phosphorylation (OXPHOS) primarily to meet their energy requirements, while under hypoxic conditions, they produce large amounts of lactate and limited ATP through anaerobic glycolysis. In Otto Warburg’s study, [37] cancer cells were shown to obtain energy through glycolysis even under aerobic conditions. After CRC development, altered glycolysis can induce CRC resistance, proliferation and metastasis by driving bacterial colonization and various signaling pathways. The active proliferation of cancer cells requires a greater supply of energy, which results in less glycolytic energy being allocated to the TME, a competitive relationship that ultimately leads to increased tumor immunosuppression.

#### Disturbed glucose metabolism is involved in immune escape in CRC

Glucose is a well-known energy compound that plays an important role in the body’s energy supply system. It has been shown that hyperglycemia is positively associated with the incidence of CRC. [38] Dysregulated expression of phosphatidylinositol-3-kinase (PI3K), mammalian target of rapamycin (mTOR) and hypoxia-inducible factor (HIF) are three important features of tumor survival and growth and are closely associated with glycolytic disturbances in CRC. [39–41] Dysregulation of these factors occurs in the following ways: 1) In CRC, PI3K is activated by the STAT3/miR-19a/PI3K axis and the mitochondria-derived ROS/PI3K axis to promote glycolysis via the classical PI3K-AKT pathway. [42] 2) mTOR, a positive regulator of aerobic glycolysis and proliferation in CRC cells, is activated by the RAS gene (approximately 52% of CRC patients carry mutations in the RAS gene) and promotes the upregulation of glucose transporter protein 1 (GLUT1), thereby increasing glycolysis in CRC. [43] 3) In CRC, hypoxia becomes a prominent feature of the TME due to rapid and uncontrolled tumor proliferation and inadequate blood supply. Under hypoxic conditions, HIF is activated by AKT-mTOR-HIF1α regulation and promotes cellular glycolysis in the TME by upregulating the expression of glycolytic enzymes such as glucose transporter protein 3 (GLUT3), phosphofructokinase 1 (PFK1), hexokinase 2 (HK2), lactate dehydrogenase A (LDHA) and pyruvate kinase M2 (PKM2). [44] By increasing glycolysis, glycolytic intermediates can enter anabolic pathways, and the expression of several key enzymes, including elevated glucose-6-phosphate dehydrogenase (G6PD), is increased in CRC, where they stabilize HK2, thereby reducing CD8 + T-cell infiltration through hexokinase 2-mediated IκBα phosphorylation, facilitating tumor immune escape and promoting CRC progression. [45] Tumor-associated macrophages (TAMs) are the main tumor-infiltrating immune cells and are classified into M1 and M2 types. M2 macrophages promote tumor cell proliferation and invasion through the activation of Th2 responses, etc., and these findings were also confirmed in the corresponding report on CRC. [46, 47] Regarding the effects on TAMs after glycolytic reprogramming, there is a main component: Toszka Bohn et al. suggested that acidification of the tumor microenvironment of CRC due to high glycolytic activity can induce G protein-coupled receptor-dependent expression of the transcriptional repressor ICER in TAMs, leading to its functional polarization to the M2 phenotype and promoting the growth of CRC. [48] T cells are also altered following disruption of glycolysis. In CRC, Lu Y et al. reported that the glucose response transcription factor MondoA senses the amount of glucose in the Treg cell surroundings and is regulated through the MondoA-TXNIP axis. [49] Inhibition of the MondoA-TXNIP axis promotes glycolysis, which in turn promotes Th17-related inflammation by inducing hyperglycolytic Th17-like Tregs and suppressing the antitumor function of CD8 + T cells, which ultimately contributes to the development of CRC. Furthermore, activation of the Akt/mTORC1 signaling pathway in a hyperglycemic state induces an increase in autophagic flux in CRC, a condition that has an inhibitory effect on Treg glycolysis, thereby affecting T-cell survival, proliferation and function. [50] In addition, a rather interesting article recently mentioned that fasting can inhibit aerobic glycolysis and the incidence of disease in CRC. [51] These findings coincide with previous studies that have shown that a high sugar environment is positively associated with the incidence of CRC. In the clinical setting, we may be able to assess the benefits and risks of fasting and develop a qualified and effective daily food or energy intake chart for effective cancer control.

#### Lactic acid is involved in the immune escape of CRC cells

**Fig. 3:**
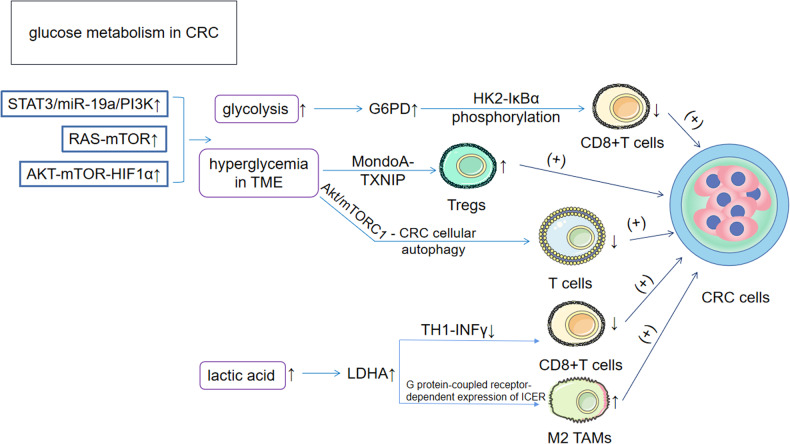
Effects of glucose metabolic reprogramming on CRC. 1) The rise in three proxies marked the upregulation of glycolysis in CRC and hyperglycaemia in CRC, both of which resulted in worsening colorectal cancer by affecting T cells through the rising G6PD, MondoA-TXNIP signalling pathway, and Akt/mTORC1-CRC cellular autophagy pathway, respectively. 2) Lactate metabolism is upregulated in CRC, and upregulation of LDHA downregulates CD8 + T cells through downregulation of TH1-INFγ, and also enables polarization of TAMs to M2 TAMs through G protein-coupled receptor-dependent expression of ICER. G6PD glucose-6-phosphate dehydrogenase; LDHA lactate dehydrogenase A Gene; ICER inducible cAMP early repressor.

Lactate, one of the products of glycolysis, is an important fuel in human metabolism and is involved in the tricarboxylic acid (TCA) cycle. As complex immunomodulatory molecules, they reprogram immune cells, exert effector functions that control innate and adaptive immune cells and promote tumor development by recruiting and inducing the activity of immunosuppressive cells and other molecules involved in cancer development. [52] CRC cells are among the most active cancer cells that produce lactate. [53] The production of lactate in aerobic glycolysis is achieved mainly through the lactate dehydrogenase family. Furthermore, LDHA plays a role in CRC in the following ways: 1) In the TME of CRC, LDHA causes an increase in lactate, which accumulates intracellularly and interferes with Th1 cell energy metabolism, and the increased concentration of lactate downregulates nuclear factors that activate T cells in Th1 cells, thereby reducing IFNγ production, leading to CD8 + T-cell inactivation and suppressing tumor immune surveillance [54]; and 2) downregulation of LDHA inhibits lactate secretion by tumor cells, thereby inhibiting the M2-like polarization of TAMs and counteracting CRC progression. [55] Hypoxia-inducible factor 1-alpha (HIF-1α), which is elevated in CRC patients and is associated with poor prognosis, [56] promotes glycolysis and lactate production by inducing the expression of lactate dehydrogenase B (LDHB), a target gene involved in glycolysis (a glycolytic enzyme), [57] upregulates PD-L1 blockade and ultimately induces immune escape. [52] Additionally, reduced lactate dehydrogenase D (LDHD) expression may lead to lactate accumulation and result in immune cell infiltration and PD-L1 inhibition. Furthermore, in mice with the CRC cell line CT26, one of the m6A demethylases, AlkB homolog 5 (AlkBh5), affects lactate secretion and regulation in cancer cells via Mct4/Slc16a3, which in turn leads to a reduced abundance of myeloid-derived suppressor cells (MDSCs) and Tregs in the TME, producing a positive outcome for the prognosis of CRC. [58] In conclusion, improving immune cell enrichment or immunosuppression in CRC by limiting lactate dehydrogenase may be a new therapeutic approach **(**Fig. 3**)**.

### Effect of lipid metabolism on the immune microenvironment in CRC

Lipid metabolism, as an important source of energy and one of the key components of cell membranes, is closely linked to cell proliferation and immune function. A high-fat diet can promote CRC progression by modulating the intestinal flora, [59] and a high-fat diet is also associated with the TME in CRC patients and can promote colorectal cancer development by impairing the infiltration and function of CD8 + T cells and inhibiting their antitumor immune cell function. [60] Taken together, these findings suggest that reprogramming of lipid metabolism occurs widely in cancer as a hallmark behavior, influencing the immune microenvironment and prognosis of CRC. In addition, in CRC, abnormalities in the metabolism of the three major lipids (fatty acids, cholesterol and phospholipids) involve several key factors, and their role in the immune microenvironment of CRC tumors should not be underestimated. The abnormal manifestations of the various types of lipids involved in the reprogramming of lipid metabolism are closely related to CRC and the associated immune microenvironment.

#### Fatty acid metabolism disturbances are involved in changes in the immune microenvironment in CRC

Fatty acids are incredibly important agents in the study of biology and human nutrition, and they also play safe and effective anti-inflammatory and immunomodulatory roles in the human body. Lipid droplets (LDs) are the main site of intracellular fat storage, and the accumulation of lipid droplets in vivo is often a result of the high energy demands of rapidly proliferating tumor cells; this process is no exception in CRC. [61, 62] LDs play a role in tumor progression in the following ways: 1) LDs are transferred to the LD membrane by adipose triglyceride lipase (ATGL) and hormone-sensitive lipase (HSL) and cleave FAA from stored triglycerides, which are a source of fatty acids for TAM polarization. Thus, LD aggregation contributes to the M2-like polarization of TAMs and promotes tumor development. [63] 2) Calreticulin (CRT) is a multifunctional protein in the endoplasmic reticulum that is involved in tumor formation and progression by promoting dendritic cell (DC) maturation. The accumulation of LDs can block CRT exposure, thereby preventing DC maturation, delaying DC functional activation and promoting tumor progression [61]; and 3) the accumulation of LDs can deplete CD8 + T cells, aid tumor immune escape and subsequently accelerate CRC progression. [61] Having seen these results, we need to explore in more depth the signaling pathways involved in the immune effects caused by lipid droplets. Additionally, prostaglandins are a type of unsaturated fatty acid that play an important role in the development of CRC [64] and regulate the function of various immune cells by binding to downstream receptors to affect the TME. Prostaglandin E2 (PGE2) binding to prostaglandin E receptor 1 (EP1) upregulates Fas ligand (FasL), [65] which in turn mediates a decrease in CD8 + T-cell activity through the Fas–FasL mechanism, thus allowing CRC cells to evade T-cell immunosurveillance. Interestingly, in CRC, Fas protein levels are usually low, which leads to immune escape in tumors. [66] EP4 is the main factor involved in the immune efficacy of prostaglandins, and when combined with PGE2, it promotes the activation of M2-type activated TAMs. [67] In fatty acid metabolism, the carnitine palmitoyltransferase CPT1A, a key rate-limiting enzyme for fatty acid β-oxidation, is highly expressed in CRC, [68] and its upregulation causes an increase in fatty acid oxidation (FAO), which enhances the function of MDSCs, which in turn suppresses T-cell immunity and promotes malignant cell proliferation and migration to promote tumor growth. [69] In addition, FAO can also promote tumor growth by upregulating PD-1 and carnitine palmitoyltransferase IA to limit Teff cell activity, thereby downregulating INF-γ production and protecting CRC cells from death. [70] FAO also drives the maturation of macrophages capable of expressing CD206, which is induced by tumor cells during macrophage infiltration and is associated with poor prognosis. [63] Fatty acid metabolism is a major metabolic process, and when considering future treatments for CRC, there is good reason to believe that a metabolic approach targeting multiple enzymes and branches could be very effective against tumors. Fortunately, one of the important fatty acid metabolism representatives summarized in this article, prostaglandins, and the key rate-limiting enzyme of FAO, carnitine palmitoyltransferase, are relatively simple to control. Some treatments for these two key factors have been reported in the literature. The next step is to combine these methods in the clinical setting. Surprisingly, recent studies have shown that the gut microbiome may have profound effects on the metabolic reprogramming of colorectal cancer. The gut microbiome can generate short-chain fatty acids (SCFAs) through two processes: 1) the breakdown of dietary fiber by fiber-degrading enzymes into shorter carbon chains for fermentation and 2) the interplay between different microbial species through mutual metabolic interactions. [71, 72] SCFAs, important weapons in the broad regulatory role of the intestinal flora, can regulate immune cell function and apoptosis and inhibit tumor growth by activating GPCR (G protein-coupled receptor) signaling pathways, such as those involving GPR43. [73, 74] In addition to aiming at traditional fatty acids, we have a new target for targeted therapy.

#### Effect of cholesterol and its metabolites on the immune microenvironment of CRC

Cholesterol, which is involved in cell membrane construction, hormone synthesis, vitamin D production and bile acid secretion, is essential for normal cellular and systemic functions. Cholesterol is capable of inducing immune cell initiation and activation by itself and its metabolites, and its main product, bile acids, also function as a regulator of the innate immune system of the intestine. In CRC, cholesterol undergoes metabolic reprogramming and undergoes synthetic upregulation, a process that is dependent on the expression of SREBP2 by the PI3K/AKT/mTOR axis. [75, 76] In CRC, cholesterol can induce macrophage infiltration by inhibiting AMPKα activity in macrophages, leading to the significant production of mitochondrial reactive oxygen species (ROS), which in turn activates NLRP3 inflammatory vesicles. [77, 78] Subsequently, these vesicles secrete CCL5 and promote immune escape of CRC cells via the p65/STAT3-CSN5-PD-L1 pathway. In addition, cholesterol in the immune microenvironment induces T-cell proliferation, migratory failure and apoptosis by upregulating the expression of PD-1 and 2B4 immune checkpoints on CD8 + T cells, which is a process that CRC cells exploit to evade T-cell immune surveillance. [79] The production of bile acids is the main route of cholesterol metabolism, and in CRC, deoxycholic acid (DCA) is elevated and plays an important role in regulating the ecology of the tumor immune microenvironment: 1) DCA upregulation upregulates EGFR through the PI3K/AKT pathway, which is closely related to CD8 + T-cell function [80–82]; 2) DCA inhibits p53 through ERK1/2 activation, inhibiting p53 and decreasing the likelihood of exerting immune effects in the TME, thereby accelerating tumor progression [36, 83]; and 3) DCA can upregulate β-linked proteins, allowing regulatory T cells to differentiate in a proinflammatory direction, which in turn leads to disease progression. [84] Cholesterol and its metabolite DCA are clear targets in drug therapy, and statins and cholesterol uptake modifiers are already in use; however, the bile acid pathway in CRC needs to be tightly controlled with more effective drugs.

#### Phospholipids are involved in the immune regulation of CRC

**Fig. 4:**
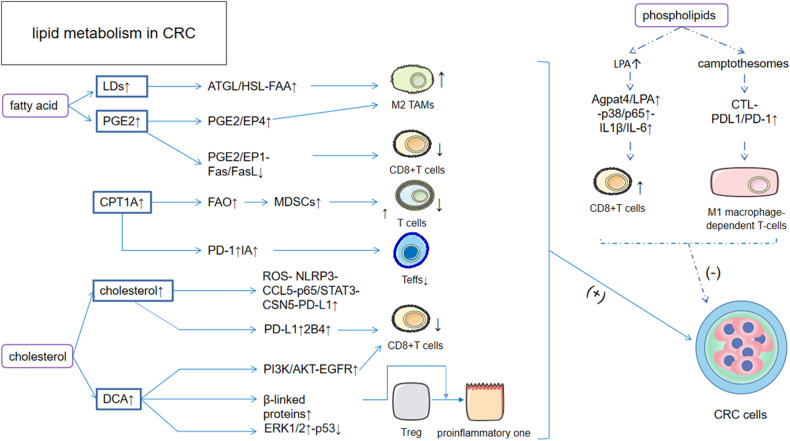
Effects of lipid metabolic reprogramming on CRC. 1) In fatty acid metabolism, LDs and PGE2 are upregulated through ATGL/HSL-FAA, PGE2/EP4 and PGE2/EP1-Fas/FasL to upregulate M2 TAMs and downregulate CD8 + T cells. And upregulation of CPT1A, a key enzyme in fatty acid metabolism, also downregulates T cells and Teffs in two different ways. In sum, the end result of fatty acid metabolism contributes to the immune escape of CRC. 2) Upregulation of cholesterol itself activates the ROS- NLRP3-CCL5-p65/STAT3- CSN5-PD-L1 pathway to directly promote cancer cell growth, and in another way downregulates CD8 + T cells through the rise of PD-L1 and marker 2B4. This is followed by a rise in its product DCA, which in turn downregulates CD8 + T cells, p53 and converts regulatory T cells to proinflammatory one through upregulation of beta-linked proteins in three ways, all of which ultimately promote CRC development. 3) Phospholipids upregulate CD8 + T cells via agpat4/LPA/p38/p65-IL1β/IL-6 upregulation; on the other hand, phospholipids also upregulate M1 macrophage-dependent T-cells via camptothesomes. ultimately phospholipids act as heterodimers in metabolic reprogramming to inhibit CRC. LDs lipid drops; PGE2 prostaglandin E2; CPT1A carnitine palmitoyltransferase 1 A; ATGL adipose triglyceride lipase; HSL hormone-sensitive lipase; DCA deoxycholic acid; LPA lysophosphatidicacid.

Phospholipids are essential in mammalian cell biology because they provide both a permeability barrier and substrates for lipid-mediated synthesis. In recent years, the role of lysophosphatidic acid (LPA) in the development of CRC has become a hot topic of research, with LPA acting through G protein-coupled receptors. [85] 1-Acylglycerol-3-phosphate O-acyltransferase 4 (AGPAT4) is a major regulator of LPA in CRC cells, and the Agpat4/LPA axis can stimulate M1-like macrophage-dependent T-cell activation, which is marked by increased IL1β and IL-6 levels via the p38/p65 pathway, by acting as a CRC suppressor. [86] In addition, the role of sphingomyelin-derived camptothecin nanovesicles (camptothesomes) in CRC has been investigated, and the following has been revealed: 1) Camptothesomes are powerful immunogenic cell death (ICD) inducers that can help disrupt the immunosuppressive TME and initiate T-cell-mediated adaptive immune control of CRC progression; and 2) Camptothesomes enhance PD-L1/PD-1 blockade via the cytotoxic T-cell (CTL) response to counteract CRC. [87] Phospholipids play an irreplaceable role in lowering blood lipid cholesterol and promoting fat metabolism and reportedly impede the metastasis of CRC to some extent; therefore, phospholipids may be the drug of choice when considering treatment strategies for CRC **(**Fig. 4**)**.

### Summary and outlook

Under normal conditions, the body maintains the functions and stability of the internal environment through three major metabolic pathways, which are reprogrammed after the onset of cancer and are involved in the alteration of the tumor immune microenvironment and the corresponding cellular immune efficacy. In CRC, alterations in amino acid metabolism, glucose metabolism and lipid metabolism lead to altered CRC cell proliferation and growth through changes in metabolic patterns and in the TME. In this paper, we review the impact of alterations in these three major metabolically important factors on the immune microenvironment of CRC patients, and we also discuss the effects of altered metabolism on different types of immune cells. We hope that this review will provide a basic and systematic summary of the field and new research directions for the study of the immune microenvironment and related therapies in CRC **(**Table 1**)**.

**Table 1:** The effects of metabolism on the immune microenvironment of colorectal cancer.

Metabolisms and metabolites	Biological effects to immune	Metabolites/enzymes involved	Roles of the metabolisms/enzymes	Results of related factors	Affected immune factors in TME	Effectiveness for CRC
amino acid metabolism						
glutamine	substrating for the proliferation, activation and efficacy of immune cells	glutamine↑	enhancing CRC cells survival and proliferation	Acetate-TCA↓	CD8+T ↓(17, 18)	promoting immune escape in CRC
Tryptophan	nutritionally essential amino acids	kyn↑	enhancing the invasiveness of CRC cells	kyn-AHR-CXCL5↑	M2 TAMs↑(27, 28)	helping CRC with immune evasion
				kyn-FOXP3↑	Tregs↑(29,30)	helping CRC with immune evasion
				TOX	CD8+T ↓(30,31)	reducing immune efficacy against CRC
		IDO↓	of CRC cells	IDO-AHR	TH17/TH1↓(33)	promoting the development of CRC
glucose metabolism						
glycolysis	energy transporter and energy supplier	PI3K↑	markers of glycolytic disorders in CRC	STAT3/miR- 19a/PI3K(39)		
		mTOR↑		RAS-mTOR(40)		
		HIF↑		AKT-mTOR-HIF1α(41)		
		G6PD↑	assisting in the metabolism of glucose	HK2-IκBα phosphorylation	CD8+T ↓(45)	promoting immune escape in CRC
		Hyperglycemia	the feature of TME in CRC	MondoA-TXNIP	Tregs↑(49)	promoting the development of CRC
				Akt/mTORC1-CRC cellular autophagy	T cells ↓(50)	promoting the development of CRC
lactic acid	an important fuel in human metabolism and TCA cycle	LDHA↑	improve lactate	TH1-INFγ↓	CD8+T ↓(54)	helping with immune escape in CRC
				G protein-coupled receptor-dependent expression of ICER	M2-TAMs↑(55)	promoting the development of CRC
lipid metabolism						
fatty acid	anti-inflammatory and immunomodulatory roles	LDs↑	main site of intracellular fat storage	ATGL/HSL-FAA↑	M2-TAMs↑(63)	promoting the development of CRC
				CRT↓	DC↓(61)	promoting the development of CRC
					CD8+T ↓(61)	helping with immune escape in CRC
			Prostaglandins↑	regulates the function of various	PGE2/EP1-Fas/FasL↓ CD8+T ↓(65,66)	helping with immune escape in CRC
		(PGE2)	immune cells by binding to downstream receptors to affect TME	PGE2/EP4↑	M2-TAMs↑(67)	promoting the development of CRC
		CPT1A↑	a key rate-limiting enzyme for fatty acid β-oxidation	FAO↑	MDSCs↑-T cells ↓(69)	promoting the development of CRC
					PD- 1 ↑IA↑-Teffs ↓-IFN-γ ↓(70)	promoting the development of CRC
					CD206 macrophages↑(63)	Poor prognosis
		gut microbiome	important components involved in intestinal homeostasis	SCFAs	GPR43↑	inhibiting the progression of CRC
cholesterol	inducing immune cell initiation and activation	cholesterol↑	immune cell initiation and activation	AMPKα↓	ROS-NLRP3-CCL5-p65/STAT3-CSN5-PD-L1↑(77,78)	helping with immune escape in CRC
				PD-L1↑2B4↑	CD8+T ↓(79)	promoting immune escape in CRC
		DCA↑	main production of cholesterol metabolism	PI3K/AKT-EGFR↑	CD8+T ↓(80-82)	promoting immune escape in CRC
				ERK1/2 ↑-p53↓	immunological effects in TME↓(36,83)	promoting the development of CRC
				β-linked proteins↑	make proinflammatory Tregs(84)	promoting the development of CRC
phospholipids	providing a permeability barrier and substrates for lipid-mediated synthesis	LPA↑	phospholipid messenger	Agpat4/LPA↑-p38/p65↑-IL1β/IL-6 ↑	M1 macrophage-dependent T-cells activation↑(86)	inhibiting the progression of CRC
		camptothesomes↑	ICD inducers	CTLs-PD-L1/PD- 1 ↑	CD8+T ↑(87)	inhibiting the progression of CRC

In this review, three major metabolic reprogrammings in colorectal cancer lead to corresponding metabolic and downstream products that affect immune components in the tumor microenvironment through up/downregulation of relevant signaling pathways or signaling factors, which in turn impact colorectal cancer progression. (Upward arrows indicate upregulation or increase, downward arrows indicate downregulation or decrease). Abbreviations: *TME* tumor microenvironment, *CRC* colorectal cancer, *TCA* tricarboxylic acid; *Kyn* kynurenine, *AHR* aryl hydrocarbon receptor, *CXCL5* C-X-C Motif Chemokine Ligand 5, *M2 TAMs* M2-type macrophages; *FOXP3*: forkhead box protein, Tregs regulatory cells, *TOX* thymocyte selection-associated HMG box protein,; *IDO* indoleamine 2,3-dioxygenase 1; *PI3K* phosphatidylinositide 3-kinases, *STAT3*: signal transducer and activator of transcription 3, *mTOR* mammalian target of Rapamycin; *HIF* hypoxia-inducible factor, *G6PD* glucose-6-phosphate dehydrogenase; *HK2*: hexokinase 2, *LDHA* lactate dehydrogenase A gene; *ICER* inducible cAMP early repressor, *LDs* lipid droplets, *CRT* calreticulin, *DC* dentritic cell, *PGE2* prostaglandin E2; *FAO* fatty acid oxidation; *MDSCs* myeloid-derived suppressor cells; *IFN-γ* interferon γ; *SCFAs* short-chain fatty acids; *AMPK* AMP-activated protein kinase; *ROS* reactive oxygen species; *EGFR* epidermal growth factor receptor; *NLRP3* NOD-like receptor thermal protein domain associated protein 3; *CCL5* C-C Motif Chemokine Ligand 5; *Agpat4* Acylglycerophosphate acyltransferase 4; *LPA* lysophosphatidic acid; *ICD* immunogenic cell death.
